# A Matter of Taste: Lineage-Specific Loss of Function of Taste Receptor Genes in Vertebrates

**DOI:** 10.3389/fmolb.2017.00081

**Published:** 2017-11-28

**Authors:** Marco Antinucci, Davide Risso

**Affiliations:** ^1^Department of Biology, University of Pisa, Pisa, Italy; ^2^Department of Genome Sciences, University of Washington School of Medicine, Seattle, WA, United States

**Keywords:** *Tas2Rs*, *TAS1Rs*, pseudogenes, sweet taste receptors, bitter taste receptors, umami taste receptors

## Abstract

Vertebrates can perceive at least five different taste qualities, each of which is thought to have a specific role in the evolution of different species. The avoidance of potentially poisonous foods, which are generally bitter or sour tasting, and the search for more nutritious ones, those with high-fat and high-sugar content, are two of the most well-known examples. The study of taste genes encoding receptors that recognize ligands triggering taste sensations has helped to reconstruct several evolutionary adaptations to dietary changes. In addition, an increasing number of studies have focused on pseudogenes, genomic DNA sequences that have traditionally been considered defunct relatives of functional genes mostly because of the presence of deleterious mutations interrupting their open reading frames. The study of taste receptor pseudogenes has helped to shed light on how the evolutionary history of taste in vertebrates has been the result of a succession of gene gain and loss processes. This dynamic role in evolution has been explained by the “less-is-more” hypothesis, suggesting gene loss as a mechanism of evolutionary change in response to a dietary shift. This mini-review aims at depicting the major lineage-specific loss of function of taste receptor genes in vertebrates, stressing their evolutionary importance and recapitulating signatures of natural selection and their correlations with food habits.

## Introduction

Pseudogenes have historically been considered genomic fossils and junk DNA, because of their classic definition of non-functional sequences of genomic DNA, originally derived from functional genes, but containing mutations or lacking promoter sequences precluding their expression (Wilde, 1986; Balakirev and Ayala, 2003). However, an increasing number of studies have shown how some pseudogenes have a function regulating gene expression (Pink et al., 2011), or being transcribed into RNA (Frankish and Harrow, 2014). Multiple studies also highlighted how pseudogenes have had a remarkable functional plasticity and a dynamic role in species' evolution (Bekpen et al., 2009; Korrodi-Gregório et al., 2013; Risso et al., 2014): chemosensory genes, in particular, have been characterized by a succession of birth-and-death processes in different lineages (Dong et al., 2009a,b). The aim of this mini-review is to provide an integrative view of the evolutionary history of taste receptor genes, with a focus on the main gene losses that have occurred in different lineages, pinpointing the genetic and biological factors driving these episodes.

## Evolution of taste receptors

Vertebrates can perceive at least five different taste qualities, each of which is thought to have evolved to face a challenge or play a specific role in species' evolution. Bitter taste, for instance, likely occurred as a defensive and protective mechanism to prevent species from ingesting potentially toxic and dangerous foods. Conversely, sweet foods are naturally attractive and accepted, since sugars serve as the main energy source for animals. Umami senses amino acids in proteins representing the taste of monosodium glutamate, naturally-occurring in meats, vegetables and fermented products. Salty and sour taste are additional protective mechanisms aimed at assuring internal sodium or acid-base balance, respectively. Genes encoding taste receptors for different tastes, as depicted below, are highly diversified in terms of both inter- and intra-specific conservation and differences (Bachmanov et al., 2014). Although most authors primarily focused on vertebrates, a few studies highlighted the presence of a large GPCR gene family in *Drosophila* named Gustatory Receptor (GR) which shows a differential expression in feeding-related tissues and confers a fine taste sensitivity to a broad range of alkaloids and other bitter compounds (Chapman et al., 1991; Glendinning and Hills, 1997; Clyne et al., 2000).

### Bitter taste

**Figure 1:**
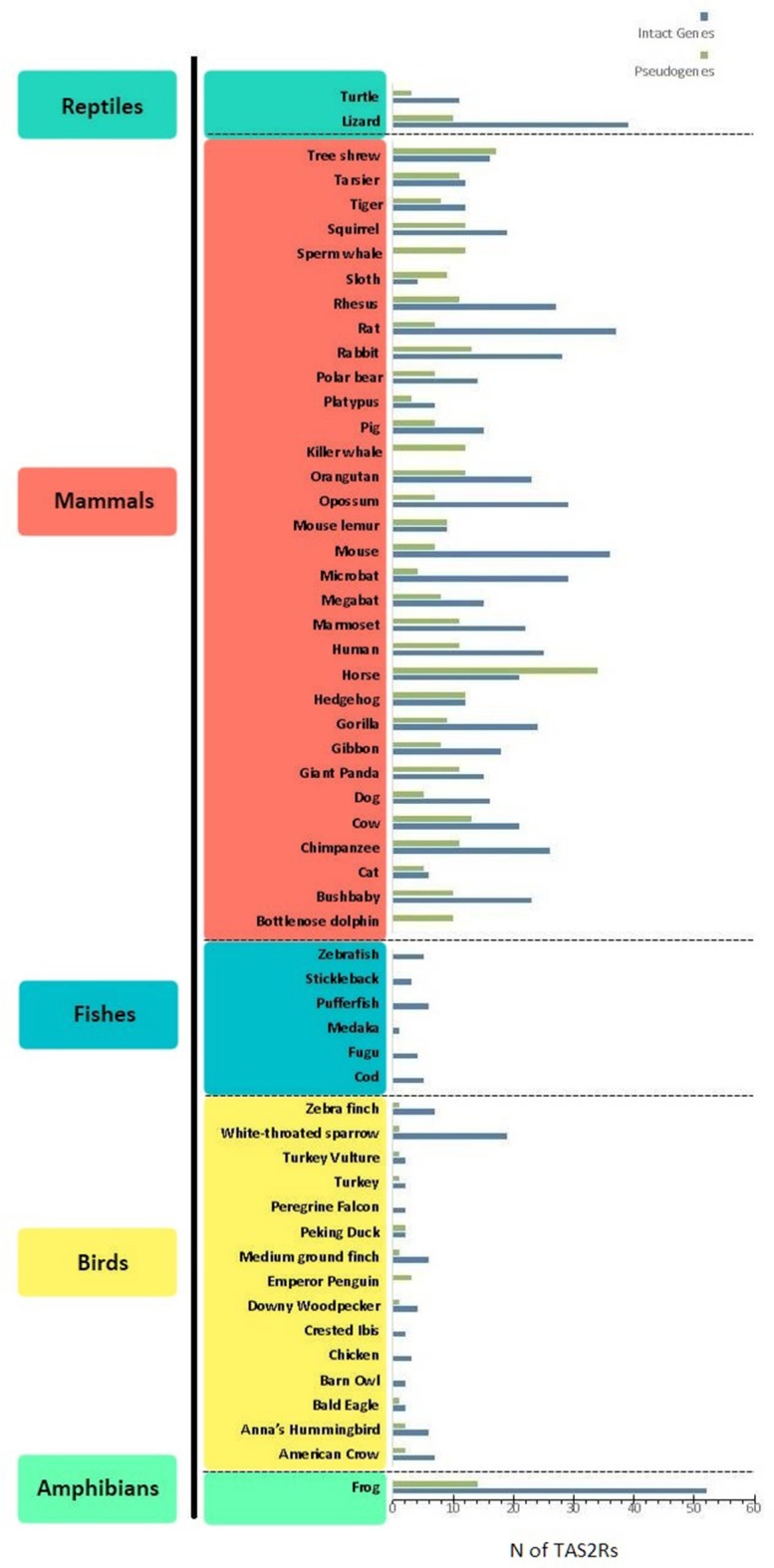
Distribution of bitter taste receptor genes (blue) and pseudogenes (green) (i.e., *TAS2Rs*) among different lineages. Data were taken from Dong et al. (2009b), Shi and Zhang (2009); Jiang et al. (2012); Li and Zhang (2013); Wang and Zhao (2015), and Liu et al. (2016). When different references indicated different numbers of *TAS2R* genes, the highest estimate was considered.

Bitter taste is recognized by receptors encoded by the *TAS2R* gene family (Adler et al., 2000). These genes are expressed in type II taste bud cells, lack introns, are relatively small (~1,000 bp) and have a short extracellular N-terminus. *TAS2R* products consist of G protein-coupled receptors (GPCRs), seven-transmembrane-domains-proteins that detect and signal neurotransmitters and hormones, among other stimuli. In particular, the binding of bitter compounds to *TAS2R* receptors activates heterometric GTP-binding proteins, consisting of a taste-selective Gα subunit (i.e., α-gustducin) and its βγ and β3γ13 partners (McLaughlin et al., 1992; Huang et al., 1999). This initiates a phosphoinositide pathway that elevates cytoplasmic Ca^2+^ and depolarizes the membrane via cation channels, resulting in the intracellular release of the taste bud transmitter ATP, which conveys signals through gustatory nerves to the brain (Chaudhari and Roper, 2010). Studies on bitter perception highlighted a differential ability to detect the bitter molecule phenylthiocarbamide (PTC) showing that this trait is linked to two haplotypes on *TAS2R38* producing the two phenotypes “taster” and “nontaster” (Kim et al., 2003). Further studies on the 3D structure of *TAS2R38* showed how PTC and its analogous molecule 6-n-propylthiouracile (PROP) can form hydrogen bounds in the transmembrane domain of the “taster” form while this bond is not formed in the “nontaster” one (Tan et al., 2012). The number of *TAS2R* genes and pseudogenes varies greatly among different vertebrate species, spanning from 0 in the bottlenose dolphin to 52 in the western clawed frog (Figure 1). A notable interspecific variation can be observed when comparing different classes of animals: mammals (*n* = 3–34) and reptiles (*n* = 3–10) for example, have a higher number of pseudogenes when compared to birds (*n* = 0–3) and fishes (*n* = 0). The limited amount of studies analyzing the *TAS2R* genes repertoire in amphibians precludes the possibility of making general considerations on this group. Further studies analyzing multiple amphibian species are therefore necessary to better comprehend the evolutionary history of these genes. Similarly, the number of functional *TAS2Rs* differs among different classes: *n* = 0–37 in mammals, *n* = 0–19 in birds, *n* = 52 in frogs, *n* = 1–6 in fishes and *n* = 11–39 in reptiles (Dong et al., 2009a; Shi and Zhang, 2009; Jiang et al., 2012; Li and Zhang, 2013; Baldwin et al., 2014; Wang and Zhao, 2015; Liu et al., 2016). Humans occupy an intermediate position, possessing 25 functional *TAS2Rs* and 11 pseudogenes mapping to chromosomes 5, 7, and 12 (Adler et al., 2000; Go et al., 2005). This remarkable variation of *TAS2R* repertoires among vertebrates has been explained by a birth-and-death model involving a complex evolution of this family, made of gene expansions, contractions, deletions, and duplications in different lineages in response to environmental changes (Shi et al., 2003; Shi and Zhang, 2005; Go, 2006; Roudnitzky et al., 2016).

### Sweet and umami taste

Sweet and umami tastes are also recognized by GPCRs that activate a signal transduction cascade through α-gustducin. However, sweet and umami GPCRs are encoded by the *TAS1R* gene family, with genes bigger in size, containing introns and a large N-terminal extracellular domain (Chen et al., 2009). This large extracellular domain is composed of two additional domains: a Venus Flytrap Domain and a cysteine-rich domain. The first is named after the carnivorous plant (i.e., *Dionaea muscipula*) as its structure can switch between open and closed conformations. The cysteine-rich domain links the first domain to the transmembrane ones and could facilitate the binding of allosteric proteins (Pin et al., 2003). Both sweet and umami receptors are heterodimers formed by two members of the *TAS1R*s gene family: the receptor encoded by the combination of *TAS1R1*+*TAS1R3* genes senses umami compounds, while the one encoded by *TAS1R2*+*TAS1R3* is activated by sweet substances (Nelson et al., 2001). A thorough research of the heterodimer *TAS1R2*+*TAS1R3*, showed how its combinations of closed-open conformations could host sweet molecules. The authors concluded that, in order to bind low molecular-weighting sweeteners, at least one monomer has to be in the open configuration. The simultaneous binding on both monomers, instead, can take place only with a combination of a closed and an open monomer, bounded to a small and a large sweetener respectively (Morini et al., 2005). This latter observation is consistent with sweetener synergy, where the action of a sweetener upon the receptor is augmented in the presence a second sweetener; moreover, the authors found that sweet proteins can bind an external portion of the receptor other than the Venus Flytrap. Additionally, sweet and umami receptors are highly conserved among different species: most vertebrates have only three receptors sensing sweet and umami tastes (i.e., *TAS1R1, TAS1R2*, and *TAS1R3*) and this configuration rarely changes, dating the divergence of these genes before the separation of teleost fishes and tetrapods, around 400 million years ago (Ishimaru et al., 2005; Shi and Zhang, 2005). However, some notable exceptions represented by lineage-specific changes in number of *TAS1R* genes, exist and will be discussed in a separate section of this mini-review.

### Salty and sour taste

Unlike bitter, sweet, and umami, the receptors responsible for salt and sour perception are not well-known. However, candidates have been identified for both tastes: studies have shown how salt and sour taste sensations are presumably not detected through GPCRs like the other tastes, but through ion channels. The amiloride-specific and sodium-specific epithelial sodium channel (ENaC) has been proposed as a candidate for salt taste perception in vertebrates (Heck et al., 1984). This membrane-bound ion-channel permeable to Na^+^ is a heteromer constituted by four subunits named α, β, γ, and δ and encoded by the closely-linked *SCNN1A, SCNN1B, SCNN1G*, and *SCNN1D* genes. These subunits are organized in two transmembrane domains, forming the pore through which Na^+^ can enter the intracellular environment. Modeling studies propose ligand binding-induced rotational movements of the pore altering the position of residues and enabling cation entrance (Kashlan and Kleyman, 2011). Genes encoding for them were found in both tetrapods and the coelacanth, indicating their ancient evolutionary origin (Giraldez et al., 2012). Many ion channels have also been proposed as sour taste perception mediators, even though the genetic basis of the perception of this taste are still poorly known. However, two transient receptor potential (TRP) channels, PKD1L1 and PKD2L3 (polycystic kidney disease-1- and −3-like), have been identified as potential candidates for sour transduction (Ishimaru et al., 2006). TRP structure comprises six transmembrane domains with a pore loop situated between the fifth and the sixth domains (Venkatachalam and Montell, 2007). PKD1 and PKD2 possess a domain with an apical pore at the top of such TRP structure, surrounded by negatively charged residues possibly drawing cations to this molecular entrance. Movements of the transmembrane helices are involved in the opening of the pore loop and thus in the activation of the receptor (Grieben et al., 2017). Although these remarkable efforts have been made to identify potential candidates for the transduction of salty and sour taste, a deep understanding of the molecular basis of these tastes is still lacking and further studies are therefore necessary to better support the physiological and genetic evidence on such candidate genes.

## Lineage-specific gene losses

Although most vertebrates can detect the same five basic tastes, some have lost particular tastes along their evolutionary paths. For most cases, these lineage-specific losses have occurred in response to specific dietary changes, feeding ecologies or changes in the environment. This complex network of co-evolution between organisms and niches is explored by the niche construction theory, where modifications of species' niches cause diverse evolutionary constraints and ecological inheritances (Odling-Smee et al., 1996). Table 1 recapitulates the major lineage-specific taste losses occurred in different vertebrate lineages, together with their genetic signatures, as a consequence of the intimate correlation between taste behavior, feeding ecology and taste receptor function. Most of the taste losses regard sweet or umami tastes, associated with the pseudogenization of *Tas1r2* and either *Tas1r1* or *Tas1r3* respectively: in particular, the loss of sweet taste perception for obligate carnivores is hypothesized to have been the result of their meat-only diets. Other animals have lost more than one taste quality, being adapted to swallowing their food whole: this has been associated with the loss of bitter *Tas2rs* and sour *Pkd2l1* genes (Beauchamp and Jiang, 2015; Zhao et al., 2015). In this paragraph, we explore some of the most exemplifying cases of taste losses, focusing on the genetic basis of these episodes.

**Table 1:** Correlation of pseudogene and taste losses in different lineages.

**Species**	**Pseudogene/Absent**	**Taste loss**	**Reference(s)**
**MAMMALS**			
African lion (*Panthera leo krugeri*)	*Tas1r2*	Sweet	Li et al., 2009
Asian small-clawed otter (*Aonyx cinerea*)	*Tas1r2*	Sweet	Jiang et al., 2012
Baleen whales (Mysticei, five species)	*Tas1r1, Tas1r2, Tas2rs, Pkd2l1*	Sweet, umami, bitter, sour	Feng et al., 2014
Banded linsang (*Prionodon linsang*)	*Tas1r2*	Sweet	Shi and Zhang, 2005
Bats (31 species)	*Tas1r1*	Umami	Zhao et al., 2010b, 2011
Bottlenose dolphin *(Tursiops truncatus*)	*Tas1r1, Tas1r2, Tas2rs, Pkd2l1*	Sweet, umami, bitter, sour	Jiang et al., 2012; Feng et al., 2014
California sea lion (*Zalophus californianus*)	*Tas1r2, Tas1r3*	Sweet, umami	Jiang et al., 2012
Cheetah (*Acinonyx jubatus*)	*Tas1r2*	Sweet	Li et al., 2005
Domestic cat (*Felis catus*)	*Tas1r2*	Sweet	Li et al., 2005
Fossa (*Cryptoprocta ferox*)	*Tas1r2*	Sweet	Jiang et al., 2012
Fur seal (*Arctocephalus australis*)	*Tas1r2*	Sweet	Jiang et al., 2012
Giant panda (*Ailuropoda melanoleuca*)	*Tas1r1*	Umami	Li et al., 2010; Zhao et al., 2010a
Pacific harnor seal (*Phoca vitulina*)	*Tas1r2*	Sweet	Shi and Zhang, 2005
Red panda (*Ailurus fulgens*)	*Tas1r1*	Umami	Hu et al., 2017
Spotted hyena (*Crocuta crocuta*)	*Tas1r2*	Sweet	Shi and Zhang, 2005
Tiger (*Panthera tigris*)	*Tas1r2*	Sweet	Li et al., 2005
Toothed whales (Odontoceti, seven species)	*Tas1r1, Tas1r2, Tas2rs, Pkd2l1*	Sweet, umami, bitter, sour	Feng et al., 2014
Vampire bats (genus *Desmodus*)	*Tas1r2, Tas1r3*	Sweet, umami	Zhao et al., 2010b, 2011
**BIRDS**			
Birds (ten species)	*Tas1r2*	Sweet	Baldwin et al., 2014
Chicken (*Gallus gallus domesticus*)	*Tas1r2*	Sweet	Shi and Zhang, 2005
Penguins (three species)	*Tas1r2, Tas1r3, Tas2rs*	Sweet, umami, bitter	Zhao et al., 2015
Turkey (genus *Meleagri*)	*Tas1r2*	Sweet	Feng and Zhao, 2013
Zebra finch (*Taeniopygia guttata*)	*Tas1r2*	Sweet	Feng and Zhao, 2013
**AMPHIBIAN**			
Tongueless western clawed frog (*Xenopus tropicalis*)	*Tas1r2*	Sweet	Shi and Zhang, 2005

### Aquatic mammals

Several terrestrial mammals have returned to the water for a fully aquatic lifestyle: among these, Cetacea and Carnivores represent two orders that returned to the sea around 50 and 35 million years ago, respectively (Uhen, 2007; Jiang et al., 2012). This change in lifestyle and habitat inevitably caused more or less dramatic changes in the anatomy and behavior of these creatures, depending on the lineage. One change in common to all these lineages is their feeding behavior: this dietary switch introducing meat in their diets reduced the importance of some tastes (i.e., bitter and sweet), because of the presence of little or no bitter and sweet compounds in meat. Many species of these orders also swallow their prey whole without mastication, reducing the importance of tasting their food. In addition, the high sodium concentration in oceans masks other tastes, further decreasing the need for taste in the feeding behaviors of these animals. These behavioral observations are consistent with anatomical evidence, showing how some aquatic mammals have atrophied taste systems with few or no taste buds (Yoshimura and Kobayashi, 1997; Yoshimura et al., 2002). A survey of taste receptor genes analyzed these loci in 12 species of toothed and baleen whales and compared it to various other species (i.e., rat, dog, human). The study better defined the repertoire of taste genes in the examined species, highlighting a relaxation of selection forces since the common ancestor of whales (between 36 and 53 Ma) and showing how all members of bitter and sweet/umami gene families have been pseudogenized because of shared premature stop codons. Moreover, the authors noted how the candidate gene for sour perception (i.e., *Pkd2l1*) is a pseudogene as well, while the candidate salty taste receptor genes (*Scnn1a, Scnn1b*, and *Scnn1g*) are intact and have experienced strong purifying selection (Feng et al., 2014). Another study showed how *Tas1r1, Tas1r2*, and *Tas1r3* are also pseudogenized in the sea lion, because of deletions or nonsense mutations (Jiang et al., 2012).

### Terrestrial carnivores

Carnivores that remained on mainland also experienced taste losses during their evolutionary history. Remarkably, sweet taste was independently lost in several lineages: dietary specializations, rather than dietary switches, were likely the drivers of these events. A study on the Carnivore order with different dietary habits (i.e., obligate carnivores, omnivorous and herbivores), for example, showed how the pseudogenization of the sweet taste receptor gene, *Tas1r2*, was widespread but independent in many lineages, because of mutations disrupting its open-reading-frame. This pseudogenization is also related to animals' diets: exclusive meat eaters are in fact lacking an intact form of this gene. In addition, Jiang et al. (2012) analyzed *Tas1r2* sequences in the order Carnivora: the authors calculated the nonsynonymous-to-synonymous substitution ratio (dN/dS) finding a ratio significantly lower than one. This led to the conclusion that purifying selection may have been acting on these genes of this order. However, despite their carnivorous diet, both the Canadian otter and the ferret have an intact *Tas1r2* (sweet) receptor sequence, suggesting that additional factors may have been involved in shaping feeding strategies and gene loss/retention. Nonetheless, further studies showed how cats and felids' (i.e., tigers, cheetahs and lions) indifference toward sugar can be accounted by the pseudogenization of *Tas1r2*, reflecting their eating behaviors. As obligate carnivores, cats and other felids possess a high-protein diet, with little carbohydrates and no simple sugars contained in plants (Li et al., 2005, 2009). Conversely, despite their herbivorous diet, horses miss *Tas1r2* (Zhao et al., 2010b). Thus, the correlation between diet and disrupting events upon this sequence may not always be the best explanation. Nonetheless, domestic cats with a carnivore diet still retain functional bitter taste receptor genes (Sandau et al., 2015). Considering that bitter receptors are expressed in extra oral tissues (Behrens and Meyerhof, 2011a,b), selective forces could have also acted on these compartments. Further studies investigating these extra-oral bitter taste receptors will be crucial in understanding the selective forces acting in these gain-and-loss mechanisms. Another example comes from the giant panda and red panda, with specialized herbivore bamboo-based diets, have a functional copy of *Tas1r2* but a pseudogenized *Tas1r1* (encoding the umami receptor) because of insertions or deletions interrupting its open-reading-frame. This was interpreted as convergent evolution in response to adaptation to a specialized diet (Li et al., 2010; Zhao et al., 2010a; Hu et al., 2017). Unlike panda, horse and cow intriguingly retain a functional *Tas1r1* and have an herbivorous diet (Zhao et al., 2010a). Similarly, vampire bats lack a proper *Tas1r2* even though sugars are present in blood, their sole food source (Zhao and Zhang, 2012).

### Birds

An additional example of how evolution accounts for taste comes from birds, descendants from theropod dinosaurs with a carnivorous diet (Sereno, 1999). A study analyzing *Tas1r* genes in 10 bird species with different diets (i.e., insectivorous, nectarivorous, and frugivores) demonstrated how birds were lacking *Tas1r2*, while the two other *Tas1r* genes (i.e., *Tas1r1* and *Tas1r3*) were present and intact. It was also suggested that the *Tas1r2* loss likely occurred within Dinosauria, before the non-avian reptile and bird lineages split (Beauchamp and Jiang, 2015). However, the observation that hummingbirds, differently from other birds, showed a distinctive preference for nectar and sugar solutions, initially represented a paradox. This was solved by the striking finding that hummingbirds' *Tas1r1* and *Tas1r3*, responsible for umami sensitivity in other species, were repurposed through the appearance of mutations leading to the acquisition of sugar responsiveness. *In vitro* reconstructions of chicken and hummingbird chimeric T1R1/T1R3 receptors, highlighted in fact how 19 amino acids in the hummingbird's T1R3 Venus Flytrap domain are responsible of modulating sucrose and sucralose perception. Intriguingly, a response to sucrose was only detected when all 19 amino acids were tested in the same chimeric receptor. These mutations were also found to be under positive selection, suggesting a new energy source unavailable to other birds (Baldwin et al., 2014).

In addition, other birds have lost more than one taste quality (similar to aquatic mammals): penguins, for example, have lost three basic tastes along their evolutionary path. A study analyzing genomes of 16 bird species has shown how penguins lack functional *Tas1r1, Tas1r2*, and *Tas1r3*. Further analyses showed that the pseudogenization of *Tas1r1* happened in the common ancestor of all penguins, since its separation from tubenose seabirds (Zhao et al., 2015). These taste losses correlate well with penguins' feeding behaviors of swallowing food whole and their tongue structure and function, suggesting that these animals don't have a real need of perceiving the taste of their food.

### Primates

The evolutionary role of bitter taste receptors (i.e., *TAS2Rs*) for avoiding potentially toxic and harmful compounds is of particular importance when considering that even small changes in the *TAS2R* gene repertoire could affect feeding habits of different animals, considering ligand-receptor specificity (Bachmanov et al., 2014). Analysis of primates showed a higher level of pseudogenes accumulation after the separation from the common ancestor of other species, such as rodents (Go et al., 2005). Nonhuman primates differ significantly in the *TAS2R* repertoire in respect to humans, showing higher pseudogenization rates. However, a relaxation of selective constraint on human *TAS2Rs* occurred instead of positive selection (Fischer et al., 2004; Wang et al., 2004; Go et al., 2005; Risso et al., 2016). In primates, lineage-specific gene duplication and pseudogenization events played a major role in shaping the *TAS2R* gene repertoire. In particular, only eight pseudogenes are in common to more than one primate species, while the majority (*n* = 23) of these pseudogenization events occurred specifically in different lineages: five in prosimians and tupais, and 19 in anthropoidea, respectively. Interestingly, three of these pseudogenes (*TAS2R2, TAS2R62*, and *TAS2R64*) are uniquely confined to *Homo sapiens* because of a two-base deletion at codon position 160, two fixed nonsense mutations at codon positions 235 and 292 and one fixed nonsense mutation at codon position 280, respectively. In addition, the two-base deletion inactivating *TAS2R2* is polymorphic in modern human populations and archaic humans, and *TAS2R64* also became a pseudogene in the orangutan because of a different nonsense mutation, showing convergent evolution (Go et al., 2005; Perry et al., 2015; Risso et al., 2017). These lineage-specific gene losses likely reflect different responses to environmental changes, resulting from species-specific food preferences during primates' evolution (Go et al., 2005); (Liman, 2006; Hayakawa et al., 2012, 2014; Risso et al., 2017). In particular, an *in vitro* study showed how the receptors encoded by the reconstructed human-specific pseudogenes and the respective chimpanzee orthologues recognized different ligands because of interspecific amino acid changes, likely allowing the adaptation to different environments and the identification of compounds of species-specific relevance (Risso et al., 2017). Similarly, a marked diversification of the *TAS2R* repertoire in terms of whole-gene deletions, gene-conversion variations and copy number variations was identified among subspecies of chimpanzees, likely reflecting their subspecies-specific dietary habits (Hayakawa et al., 2012; Wooding et al., 2012).

## Conclusion

Maynard Olson's “less-is-more” hypothesis sees gene loss as an engine of evolutionary change, representing a common response of different lineages undergoing similar environmental shifts (Olson, 1999; Callaway, 2012). Some of the lineage-specific pseudogenization events discussed in this mini-review have been related to specific feeding behavior, dietary switches or environmental changes at both large and small scales, highlighting the plasticity and dynamism of the taste system (Li et al., 2005; Liman, 2006; Zhao et al., 2010a, 2011; Hayakawa et al., 2012; Jiang et al., 2012; Baldwin et al., 2014; Feng et al., 2014; Wang and Zhao, 2015; Risso et al., 2017). In addition, the repertoire of bitter taste receptor genes has been associated with species' feeding behaviors, correlating with the fraction of plants in their diet as an evolutionary mechanisms protecting from ingesting potentially toxic compounds (Li and Zhang, 2013; Wang and Zhao, 2015; Liu et al., 2016; Shang et al., 2017). However, birth-and-death events of taste receptor genes are not always correlated with species' feeding behaviors, where putative useless taste receptor genes are still present, making it possible to detect a taste quality that should not be present in their diet. These data suggest that there is still a gap in our understanding of the physiological function of these genes (Zhao and Zhang, 2012; Feng and Zhao, 2013; Liu et al., 2016). This encourages further genetic and behavioral studies aimed at better identifying the role of taste receptor genes and pseudogenes in different species, in order to shed light on the evolutionary mechanisms that have inactivated genes exclusively in specific lineages.

## Author contributions

MA wrote the first draft of the manuscript. DR contributed to writing and editing the manuscript and created the figures/tables.

### Conflict of interest statement

The authors declare that the research was conducted in the absence of any commercial or financial relationships that could be construed as a potential conflict of interest.
